# Cofitness network connectivity determines a fuzzy essential zone in open bacterial pangenome

**DOI:** 10.1002/mlf2.12132

**Published:** 2024-06-28

**Authors:** Pan Zhang, Biliang Zhang, Yuan‐Yuan Ji, Jian Jiao, Ziding Zhang, Chang‐Fu Tian

**Affiliations:** ^1^ State Key Laboratory of Plant Environmental Resilience, and College of Biological Sciences China Agricultural University Beijing China; ^2^ MOA Key Laboratory of Soil Microbiology, and Rhizobium Research Center China Agricultural University Beijing China; ^3^ Shenzhen Institute of Synthetic Biology, Shenzhen Institute of Advanced Technology Chinese Academy of Sciences Shenzhen China; ^4^ State Key Laboratory of Livestock and Poultry Biotechnology Breeding, and College of Biological Sciences China Agricultural University Beijing China

**Keywords:** cofitness network, pangenome, Tn‐seq

## Abstract

Most in silico evolutionary studies commonly assumed that core genes are essential for cellular function, while accessory genes are dispensable, particularly in nutrient‐rich environments. However, this assumption is seldom tested genetically within the pangenome context. In this study, we conducted a robust pangenomic Tn‐seq analysis of fitness genes in a nutrient‐rich medium for *Sinorhizobium* strains with a canonical open pangenome. To evaluate the robustness of fitness category assignment, Tn‐seq data for three independent mutant libraries per strain were analyzed by three methods, which indicates that the Hidden Markov Model (HMM)‐based method is most robust to variations between mutant libraries and not sensitive to data size, outperforming the Bayesian and Monte Carlo simulation‐based methods. Consequently, the HMM method was used to classify the fitness category. Fitness genes, categorized as essential (ES), advantage (GA), and disadvantage (GD) genes for growth, are enriched in core genes, while nonessential genes (NE) are over‐represented in accessory genes. Accessory ES/GA genes showed a lower fitness effect than core ES/GA genes. Connectivity degrees in the cofitness network decrease in the order of ES, GD, and GA/NE. In addition to accessory genes, 1599 out of 3284 core genes display differential essentiality across test strains. Within the pangenome core, both shared quasi‐essential (ES and GA) and strain‐dependent fitness genes are enriched in similar functional categories. Our analysis demonstrates a considerable fuzzy essential zone determined by cofitness connectivity degrees in *Sinorhizobium* pangenome and highlights the power of the cofitness network in understanding the genetic basis of ever‐increasing prokaryotic pangenome data.

## Impact statement

Core and accessory genes in the pangenome are hypothesized to be essential and dispensable, respectively, for prokaryote fitness under nutrient‐rich conditions. This bipartition view has been widely referenced in genomic studies but not effectively tested. By using network analysis of pangenomic Tn‐seq data of sibling *Sinorhizobium* strains under a nutrient‐rich condition, this work not only revealed a positive correlation of gene fitness categories with both gene conservation levels and network connectivity degrees but also uncovered an enrichment of both shared and strain‐dependent fitness genes in essential cellular functions, for example, translation and cell envelop biogenesis. This work highlights the importance of network rewiring in shaping the strain‐dependent fuzzy essential zone of the prokaryote pangenome.

## INTRODUCTION

In the essence of the biological species concept, reproductive isolation or, more generally speaking, independent evolution is considered to be virtually synonymous with the process of speciation^1^. For prokaryotes, the recombination rate declines with increased sequence divergence, and the number of documented species has been significantly enlarged by computing average nucleotide identity (ANI) in the scenario of alpha taxonomy in the past decades^2^, ^3^, ^4^, ^5^. However, a ubiquitous biological species concept for prokaryotes has been questioned^4^, ^5^, largely due to the fact that genetic differences between populations or species could be eroded by promiscuous lateral gene transfer events^4^, ^6^. This evolutionary dilemma regarding prokaryote species is tentatively solved by the split of pangenome into core genes shared by all relevant strains and accessory genes present in a subset of strains, which manage essential and nonessential cellular processes, respectively^7^, ^8^, ^9^. In other words, essential core genes define the species, while nonessential accessory genes confer adaptation potential in ever‐changing circumstances^7^, ^10^. It has been widely accepted that essential (ES) genes are those more conserved and irreplaceable members^11^, ^12^, which inspire the ongoing pursuit of the minimal genome for model organisms in the context of synthetic biology^13^, ^14^, ^15^. However, it remains elusive how pangenomes evolve^10^, ^16^, ^17^, ^18^, which determine the fitness of organisms in various habitats^19^.

Increasing in silico analyses of pangenome for a phylogenetic clade, usually a genus or species, support a hypothesis of adaptive evolution of pangenome at both gene and organism levels^17^, ^18^, ^20^, ^21^. Comparative transcriptomic studies in the pangenome context suggest that various accessory functions are usually integrated with the core regulation network at different extents, that is, the more conserved genes show a higher average transcription level and a higher connectivity degree in coexpression networks than those of less conserved ones^16^, ^22^, ^23^. A fuzzy essential zone, composed of strain‐specific ES genes, of pangenome can be hypothesized when different sibling strains are compared, but direct empirical evidence is still rare.

A global coexpression network shows potential crosstalk patterns between biological pathways at the expression level, while a related genetic interaction network is investigated by reverse and/or forward genetic procedures. Transposon insertion sequencing (Tn‐seq) can be used to massively characterize genes of unknown function among distantly related bacteria across dozens of growth conditions^24^, ^25^. Particularly, bacterial fitness genes involved in pathogenic or beneficial interactions with various eukaryotes have been intensively investigated for a single strain^26^, for example, antibiotic resistance genes of human pathogens^27^, ^28^, ^29^, and colonization determinants of human pathogens^30^, gut symbionts in honey bees^31^, plant symbionts^32^, ^33^, plant growth promotion bacteria^34^, ^35^, ^36^, and plant pathogens^37^, ^38^. Several Tn‐seq analyses have been performed on two or more sibling pathogenic strains to define a core set of ES genes or condition‐dependent ones^28^, ^29^, ^39^, aiming for identifying novel drug targets. Genes participating in the same biological processes tend to genetically interact with common sets of other genes within distinct but related pathways, leading to the emergence of strongly correlated genetic interaction profiles across a wide array of genetic backgrounds. The exploration of genetic interaction networks in model organisms has been a longstanding approach to unveil functional associations between genes or their corresponding gene products^40^, ^41^. The cofitness network^42^, which represents a kind of genetic interaction network, adopts a construction method similar to the coexpression network, except that it uses the fitness values of genes for different growth conditions. However, co‐essentiality network and strain‐dependent network rewiring have not been well‐studied in a pangenome context. A related term, “network rewiring”, referring to the inherent reorganization of interactions between biological components due to conditional changes, has become widely adopted^43^, ^44^, ^45^, ^46^. It is a fundamental characteristic of most, if not all, biological networks. The network rewiring can have a profound impact on alterations in gene essentiality since the rewiring of interactions facilitates the integration of genes into new pathways, thereby heightening the likelihood of their engagement in crucial biological processes^46^, ^47^. Thus, examining genetic network rewiring within a single strain helps us understand how that strain copes with environmental fluctuations while exploring genetic network rewiring among sibling strains can provide insights into pangenome evolution.

In this work, we aimed to investigate the putative fuzzy essential zone of closely related bacteria from both functional and evolutionary points of view. To this end, we characterized genes as ES, advantage (GA), disadvantage (GD), or nonessential (NE) genes for the growth of five sibling strains of *Sinorhizobium* representing one of the best‐studied bacterial genera of open pangenome^48^, ^49^. *Sinorhizobium* members, living saprophytically in soils as other rhizobia, can occasionally form nitrogen‐fixing nodules on diverse legumes such as the *Sinorhizobium* *fredii*‐soybean and *Sinorhizobium* *meliloti*‐alfalfa pairs^50^, ^51^. *Sinorhizobium* species are characterized by their similar multipartite genomes^22^, ^52^, ^53^ and diverged earlier than the innovation of legume nodules^23^, ^54^. To minimize the systematic error, the *Himar1* *mariner* transposase gene driven by a *Sinorhizobium rpoD* promoter was used in the construction of three independent mutant libraries for each strain, and Tn‐seq data from 15 independent libraries from five strains were analyzed by Hidden Markov Model (HMM)^55^, Bayesian^56^, and Monte Carlo simulation‐based methods^57^, respectively. The fuzzy essential zones identified by the most robust method were subject to cofitness network analysis and enrichment analyses of pangenome subsets and functional categories in the pangenome context of 17 *Sinorhizobium* species. This work revealed a positive correlation between gene essentiality grades with both gene conservation levels and network connectivity degrees. Core and accessory ES/GA genes showed different function enrichment profiles, while core ES/GA genes exhibited an enrichment of both shared and strain‐dependent fitness genes in essential cellular functions, for example, translation and cell envelop biogenesis. These findings highlight the importance of network rewiring in shaping the strain‐dependent fuzzy essential zone of prokaryote pangenome.

## RESULTS AND DISCUSSION

### HMM‐based method shows superior robustness against variations among Tn‐seq data from independent mutant libraries

**Figure 1:**
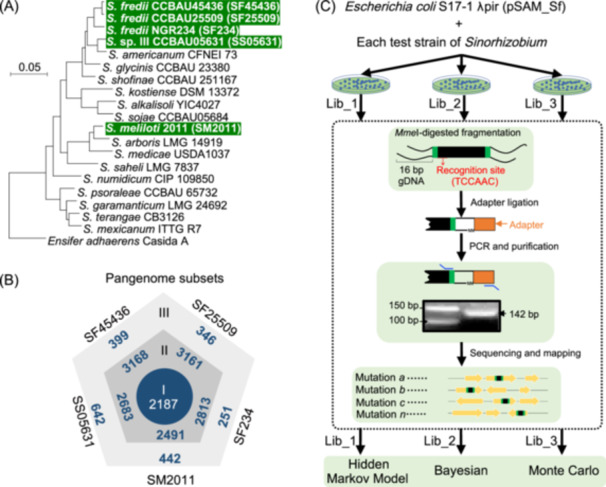
Tn‐seq analysis of *Sinorhizobium* pangenome. (A) A maximum likelihood phylogenomic tree based on the 1667 core genes shared by 19 *Sinorhizobium* strains and an outgroup strain *Ensifer adhaerens* Casida A. Bootstrap values are all 100. (B) Hierarchical divisions of core/accessory subsets for the five strains. Subset I, 2187 single‐copy protein‐coding genes shared by the five strains; Subset II, genes shared by at least two strains excluding subset I; Subset III, strain‐specific genes. (C) Workflow of the Tn‐seq analysis of *Sinorhizobium* strains. Three independent mutant libraries were constructed for individual test strains, and then mutant libraries for each strain were individually scraped and collected to do subsequent genomic DNA extraction and Tn‐Seq sample preparation for sequencing. Hidden Markov Model (HMM), Bayesian, and Monte Carlo methods were compared and used for analyzing Tn‐seq data.

**Figure 2:**
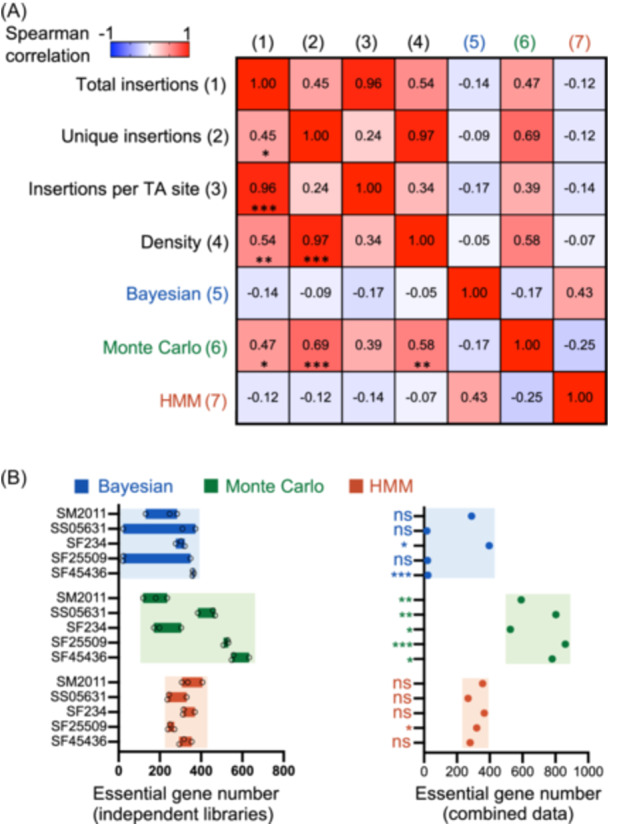
HMM method is robust for pangenomic Tn‐seq data. (A) Spearman correlation among Tn‐seq variables (from 1 to 4) and essential gene numbers identified by three methods. Significant correlation coefficient values are indicated in the bottom left of the matrix (*p < 0.05; **p < 0.01; ***p < 0.001). Only the Monte Carlo method shows a moderate but significant correlation with Tn‐seq variables, including total insertions, unique insertions, and density. (B) Essential gene number identified by Bayesian, Monte Carlo, and HMM methods. Significant difference between essential gene numbers in individual mutant libraries (left) and that in combined data (right) is indicated (one sample *t*‐test; *p < 0.05; **p < 0.01; ***p < 0.001; ns, *p* > 0.05). HMM method is robust among Tn‐seq data from independent mutant libraries and not sensitive to data size.

Stochastic differences among independent mutant libraries can affect the robustness of conclusions and have received increasing attention, particularly when comparing independent Tn‐seq studies^58^, ^59^. In this work, the *mariner* transposon known to have little sequence specificity beyond the exact insertion into thymine‐adenine dinucleotide (TA) sites^60^ was used to generate three independent mutant libraries for each test strain. This allowed systematic evaluation of insertion efficiency with available information on genome TA sites and stochastic differences among independent libraries. To assure efficient transposition in test *Sinorhizobium* strains, the *mariner*‐carrying pSAM_Bt vector developed earlier^61^ was retrofitted with a kanamycin resistance cassette from pRL1063a^62^ and the *rpoD* promoter from *S. fredii* CCBAU45436 to create pSAM_Sf (Figure S1). Three independent mutant libraries (each library with around 700,000–1,000,000 colonies) for each of five test *Sinorhizobium* strains were individually constructed in tryptone‐yeast extract (TY) medium, which is a nutrient‐rich medium routinely used for rhizobial growth^63^. These strains, including *S. fredii* CCBAU45436 (SF45436), *S. fredii* CCBAU25509 (SF25509), *S. fredii* NGR234 (SF234), *Sinorhizobium* sp. III CCBAU05631 (SS05631) and *S. meliloti* 2011 (SM2011) represent three lineages from *Sinorhizobium* (Figure 1A), and their complete genomes were obtained earlier^22^, ^23^, ^64^, ^65^. Pangenome members of the five test strains can be assigned into three subsets (Figure 1B) of the *Sinorhizobium* pangenome based on 19 strains (Figure 1A): subset I, gene homologs present in 19 *Sinorhizobium* strains; subset II, those shared by at least two strains excluding subset I; subset III, the remaining accessory genes of each strain. The 15 independent mutant pools from three independent mutant libraries were subject to an adapted version of the Tn‐seq method (Figure 1C). The number of TA sites in individual genomes ranged from 106,040 to 115,384, and 52.89%–87.09% of available TA sites were detected with insertions by the *mariner* transposon among 15 samples (Supporting Information: Data S1‐1). These insertion frequency values are all above the current threshold for good libraries (greater than 50%)^66^, and showed a strong and moderate positive correlation with the number of detected unique insertions (Spearman *r* = 0.97, *p* < 0.001) and total insertions (Spearman *r* = 0.54, *p* < 0.01; Figure 2A), respectively.

To evaluate potential library‐dependent effects on gene fitness determination, HMM^55^, Bayesian^56^, and Monte Carlo methods^57^ were used for analyzing Tn‐seq data to identify ES genes (Figure 2B and Supporting Information: Data S1‐1). The Bayesian method calculates the posterior probability of the longest consecutive sequence of TA sites lacking insertion in a gene^56^, ^66^. A considerable library‐dependent variation was observed for the number of ES, uncertain, and NE genes in SS05631 and SF25509 (Figure 2B and Supporting Information: Data S1‐1). Although the number of ES genes did not show a significant correlation with total insertions, unique insertions, insertions per TA site, or insertion density (Spearman *r* = −0.17 to −0.05; Figure 2A), individual libraries with less than 25 ES genes identified for SS05631 (above 84%) and SF25509 (above 86%) are those with the higher insertion density (Supporting Information: Data S1‐1). When data from three independent libraries were combined, SS05631, SF25509, and SF45436 with insertion density above 90% had just 17, 20, and 23 ES genes identified by Bayesian method, respectively (Figure 2B and Supporting Information: Data S1). By contrast, such inauthentic numbers of ES genes were not observed for SF234 and SM2011 with insertion density below 78% (Figure 2B and Supporting Information: Data S1‐1). Therefore, the Bayesian method may give a false negative report on ES genes when the insertion density is at a high level. Such great library‐dependent variation was not observed for the Monte Carlo method, which instead identified a considerable strain‐dependent variation in the number of ES genes (Figure 2B). This strain‐dependent variation could be greatly reduced when data from three libraries were combined (Figure 2B). This is in line with the positive correlation between the ES gene number determined by the Monte Carlo method and the number of unique insertions (Spearman *r* = 0.69, *p* < 0.001; Figure 2A), insertion density (Spearman *r* = 0.58, *p* < 0.01; Figure 2A), or total insertions (Spearman *r* = 0.47, *p* < 0.05; Figure 2A). These results are consistent with the fact that the Monte Carlo method uses “Expected” pseudo‐datasets randomly generated from the pool of obtained read counts in Tn‐seq^57^. Notably, the Monte Carlo method only defines whether a gene is ES or NE, and under the thresholds of available studies and this work^39^, ^67^, ^68^, ^69^, some GA genes can be included in the ES subset. Indeed, as shown in Figure S2, it is not rare to observe two peaks within a density distribution of fitness values for an ES subset defined by the Monte Carlo method.

The HMM method assigns gene essentiality into ES, GA, GD, or NE by calculating the likelihood of read counts in each category based on a geometric distribution^55^. As shown in Figure 2B, the HMM method was neither sensitive to stochastic variations among independent libraries as the Bayesian method did, nor severely affected by the variation of data size and insertion density (Spearman *r* = −0.14 to −0.07) as the Monte Carlo method did (Figure 2A). This is supported by the earlier observation that the HMM method can make reasonable essentiality analysis for Tn‐seq data of insertion density from dense (above 54%) to sparse (38% and 27%)^55^. By analyzing more than 70 independent studies of ES genes in diverse bacteria from DEG 15^70^, we are aware of an average minimal set of 394 ± 36 (95% confidence interval) ES genes in a bacterial species. Apparently, the HMM method performed well for both independent libraries and the combined data (Figure 2B) compared to the other two methods. Consequently, the HMM method and the combined Tn‐seq data from three libraries of individual strains (insertion density ranging from 75.5% to 91.6%) were used to define gene fitness categories: ES, GA, NE, and GD. Moreover, these fitness categories were generally supported by the gene fitness values calculated by the Monte Carlo method (Figure S2 and Supporting Information: Data S1), that is, fitness values increased in the order of ES, GA, NE, and GD.

### Gene essentiality grades positively correlate with conservation levels in *Sinorhizobium* pangenome

**Figure 3:**
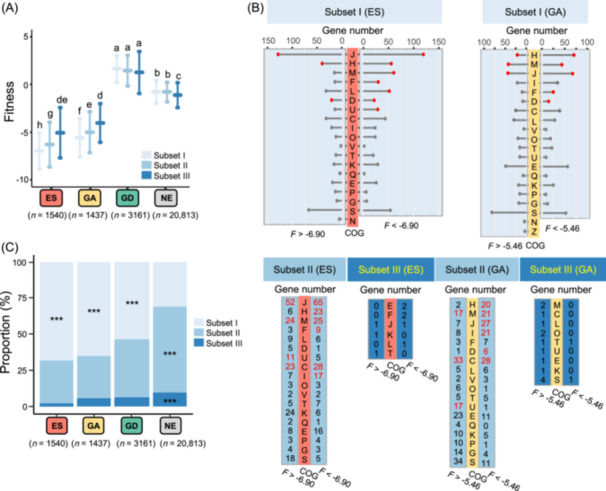
Essentiality grades correlate with conservation levels in *Sinorhizobium* pangenome. (A) Multiple comparison tests of the fitness values between genes of different conservation levels. Error bar represents SD. Fitness values are calculated by the Monte Carlo method. ES (essential), GA (growth advantage), NE (nonessential), and GD (growth disadvantage) were defined by the HMM method. Fitness values sequentially increase from subset I to II and III within ES and GA categories. (B) COG (Clusters of Orthologous Groups) enrichment analysis of ES and GA genes with fitness values (F) above or below the average fitness value of subset I (−6.90 and −5.46 for ES and GA, respectively). Gene number in a specific COG category belonging to an indicated gene subset is shown, and significant enrichment is indicated by red points or red numbers compared to the total number of genes with COG annotations in five test strains (Fisher's exact test, *p* < 0.05). D, cell cycle control, cell division, chromosome partitioning; F, nucleotide transport and metabolism; G, carbohydrate transport and metabolism; H, coenzyme transport and metabolism; J, translation, ribosomal structure and biogenesis; K, transcription; L, replication, recombination and repair; M, cell wall/membrane/envelop biogenesis; N, cell motility; P, inorganic ion transport and metabolism; S, unknown function; T, signal transduction mechanisms; U, intracellular trafficking, secretion, and vesicular transport. (C) Fisher's exact test of the proportion of genes in subset I–III (***p < 0.001). ES, GA, and GD categories are enriched with subset I genes.

We further explored the relationship between gene fitness categories and gene conservation levels. Genes within different pangenome subsets represent genes with different degrees of conservation (Figure 1B), and gene conservation levels showed a decrease in the following order: subsets I, II, and III. As expected, the average fitness values of genes, regardless of pangenome subset assignments, sequentially increased with reduced gene essentiality: ES, GA, NE, and GD (Tukey HSD, *α* = 0.05; Figures 3A and S2). Among the ES and GA genes, the average fitness values sequentially increased with gene conservation level: subset I, II, and III (Tukey HSD, *α* = 0.05; Figure 3A). Within subset I, the average fitness values for 1054 ES genes and 922 GA genes are −6.90 and −5.46, respectively. These genes with fitness values either above or below the average are enriched in COG (Clusters of Orthologous Groups) categories J (translation, ribosomal structure and biogenesis), H (coenzyme transport and metabolism), F (nucleotide transport and metabolism), L (replication, recombination and repair), D (cell cycle control, cell division, and chromosome partitioning), U (intracellular trafficking, secretion, and vesicular transport), and M (cell wall/membrane/envelope biogenesis) (Figure 3B; Fisher's exact test, *p* < 0.05). Among 455 ES genes and 437 GA genes belonging to subset II, those genes with fitness values below the subset I average (−6.90 and −5.46 for ES and GA, respectively), rather than those above the average, are more likely to have function assignment in those COG categories over‐represented in ES and GA genes of subset I (Figure 3B), and both ES and GA genes in the subset II have distinct function enrichment profiles compared to the subset I, for example, C (energy production and conversion; *p* < 0.05). Among 31 ES genes and 78 GA genes belonging to subset III, few genes have COG assignment and no significant function enrichment was identified among ES and GA genes (Figure 3B). Therefore, the function enrichment profile of ES and GA genes belonging to less conserved subsets II and III can be different from those of subset I to a certain extent, supporting a strain‐dependent rewiring of the ES/GA gene network characterized by its higher average fitness value in subsets II and III than in subset I.

The subset I was over‐represented in ES, GA, and GD, while subsets II and III were enriched in NE (Figure 3C, Fisher's exact test, *p* < 0.001; Figure S3, *Z* test, *p* < 0.01). A sequential decline of the proportion of subset I genes was observed in the order of ES, GA, GD, and NE (Figure 3C). These findings are in line with a dominant role of conserved pangenome members (subset I)^16^, ^71^, and highlight an active integration of strain‐dependent functions (subset II and III) into the core network. This ensures the growth of different sibling strains under the same nutrient‐rich condition, supporting the hypothesis of network‐based variation of cellular organisms during divergence^72^. Since new nodes and edges have been added, then how would the core network rewire?

### Cofitness network analysis reveals a fuzzy essential zone of the core genome

In addition to the strain‐dependent innovation of genes essential for growth (Figures 1B and 3), we further characterized the genes shared by five strains with network‐based methods. Pearson's correlation networks such as weighted gene coexpression network analysis have been widely used in systems biology and bacterial pangenomics^22^, ^73^, ^74^. By using an analyzing procedure similar to the gene coexpression network, the cofitness network was recently introduced in a Tn‐seq analysis of gene fitness values for *Streptococcus pneumoniae* under different conditions^42^. In this study, we constructed a cofitness network for 3284 core genes among five strains in a pangenome context (Figure S4). Briefly, the fitness values of core genes among five strains obtained by the Monte Carlo method were used to calculate Pearson's correlation coefficient. Then, the random matrix theory (RMT)‐based network approach^75^, ^76^, ^77^, ^78^ was used to define the correlation threshold (Pearson's *r* > 0.91) to construct the cofitness network. Pearson coefficient is the preferable method for normally distributed data and the default metric in gene coexpression network analyses^79^. Among the five strains, whether divided into four gene categories by the HMM method or two gene classes by the Monte Carlo method, their fitness values exhibit a normal distribution within each strain (Figure S2). In the cofitness network, a higher correlation between two genes indicates that their fitness changes consistently across different bacteria. Conversely, if a gene exhibits a strain‐dependent essentiality pattern, its degree of correlation is low. Similar to the coexpression network, a gene with identical fitness values across strains will make correlation coefficient calculation impossible. Upon examining our data, we found that none of the genes had identical fitness values across the five strains. Standard deviation (SD) for fitness values of individual core genes among five strains ranged from 0.08 to 5.45 (Figure S5A), with the GA category having the highest SD value, followed sequentially by ES, GD, and NE categories (Figure S5B).

**Figure 4:**
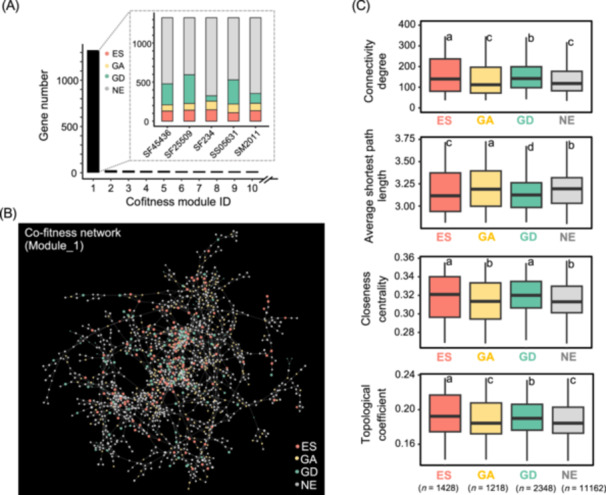
Essentiality grades correlate with network connectivity degrees of *Sinorhizobium* pangenome members. (A) The cofitness network analysis of Monte Carlo‐based fitness values (Only the top 1% of edges with the strongest relevance are included). The fitness categories are shown for Module_1. (B) The cofitness network of Module_1. The size of the filled circle is proportional to the connectivity degree in the cofitness network. The color scheme represents the fitness categories of SF45436 in (A). (C) The cofitness network analysis of connectivity degree, average shortest path length, closeness centrality, and topological coefficient. Different lowercase letters in (C) indicate significant differences between means (Tukey HSD test, adj. *p* < 0.05).

**Figure 5:**
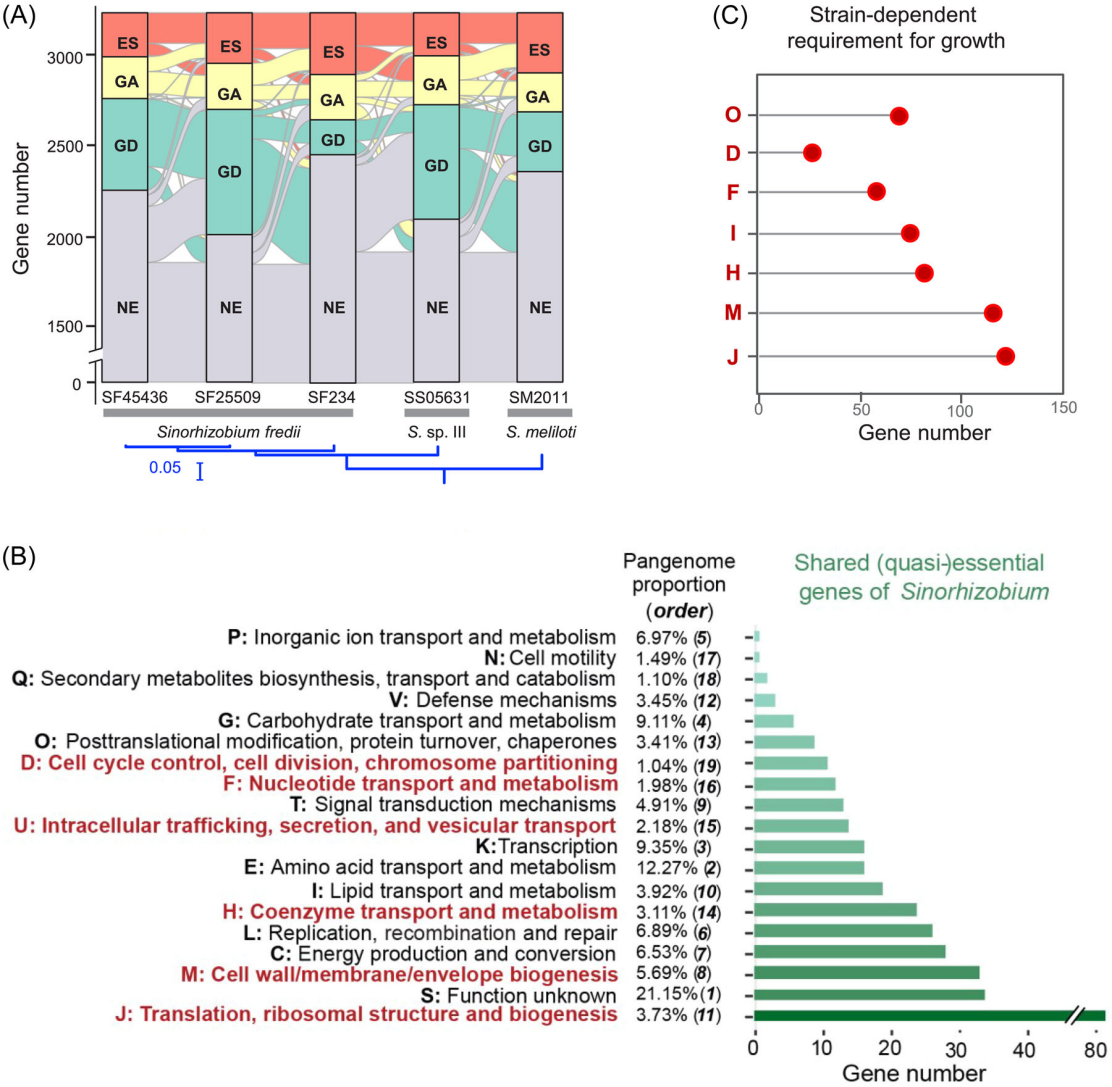
A considerable fuzzy essential zone of *Sinorhizobium* pangenome core. (A) Strain‐dependent gene essentiality of genes shared by five strains (results for subset I and II are shown in Figure S7). The Maximum Likelihood phylogenetic tree based on 3284 core genes of five test strains is shown and all branches have 100% bootstrap support. (B, C) COG enrichment analysis of shared (quasi‐)essential genes (341 essential and growth‐advantage genes) (B), and core genes showing strain‐dependent requirement for growth (C). Red in (B, C) represents* p* < 0.05 in Fisher's exact test. The proportion of each COG category and its abundance order among 27,724 genes with COG annotations in the pangenome of five test strains are shown in (B).

To better visualize the network, we constructed a network based on the top 1% of edges with the strongest relevance (involving 2757 genes), with 1325 genes belonging to the largest Module_1 that showed a strain‐dependent gene essentiality pattern (Figure 4A and highlighted in Figure S4). Genes of higher connectivity degrees (indicated by the size of the filled circle in Figure 4B; cofitness degree, hereafter) seemed to be over‐represented in the ES category (Figure 4B). When all genes of the cofitness network were analyzed (Figure S4), the ES category possessed the highest cofitness degree and topological coefficient, followed by GD and GA/NE categories (Tukey HSD test, adj. *p *< 0.05; Figure 4C). The ES and GD categories have higher closeness centrality compared to GA and NE, while the average shortest path length decreases in the order of GA, NE, ES, to GD (Tukey HSD test, adj. *p* < 0.05; Figure 4C). These network features associated with ES and GD categories are consistent with their lowest and highest fitness values, respectively (Figure 3A), implying that GD genes with significant negative fitness effects have network features more similar to those genes ES for survival compared to those NE and GA genes. When these network features were evaluated for subset I and II, respectively (Figure S6), ES, GD, GA, and NE categories can be distinguished from each other for subset I in a similar way as the whole network but less significant for subset II. For example, ES and GD categories have a similar cofitness degree, average shortest path length, closeness centrality, and topological coefficient, and GA and NE categories also have similar network features, except higher cofitness degree of GA than NE, for subset II (Figure S6). Taking together, these results imply that the cofitness network is more conserved in the ES category, and network rewiring level is higher in GD, GA, and NE categories among *Sinorhizobium* strains. Therefore, the putative stable core genome^80^ is not as “still” as expected between sibling strains. Furthermore, the strain‐dependent gene essentiality profiles of genes shared by five strains revealed that ES and GA categories intermingled with each other among test sibling strains (Figure 5A). Therefore, ES and GA genes can be collectively defined as (quasi‐)essential genes.

Collectively, there was a strain‐dependent variation in the cofitness networks (Figure 4A) of the *Sinorhizobium* core genome. A significant network rewiring was observed for the GA, GD, and NE categories (Figure 4C). These network characteristics suggest that the minimal genome^15^ can be viewed as a highly connected network, and beneficial (GA) or deleterious (GD) rewiring events are more likely to happen for those nodes with lower connectivity. In the pangenome context, the “complexity hypothesis” was coined to depict the dependency of gene horizontal transferability on the network connectivity and biological process^81^, ^82^, with the former playing a dominant role^83^, ^84^, ^85^. As many as 92% of gene families in bacteria have evidence of horizontal transfer^86^, and the observed variation in network connectivity and network rewiring level in *Sinorhizobium* core genome provides valuable pangenome evolutionary information for further synthetic biology studies^87^.

### Evolutionary and functional insights into the cofitness network of *Sinorhizobium*

Within the cofitness network of five *Sinorhizobium* strains, 71, 9, 50, and 1553 genes belonging to ES, GA, GD, and NE categories, respectively, were shared by five strains (Figure 5A and Supporting Information: Data S1‐2, S1‐3, S1‐4). When the intermingled ES and GA categories were combined, the new (quasi‐)essential category harbored 341 genes shared by five strains (Supporting Information: Data S1‐2). This value is close to the average size of a bacterial minimal genome (394 ± 36; 95% confidence interval)^70^, implying that this subset may represent the ES genome of the last common ancestor of extant *Sinorhizobium* strains. This procedure may be generalized to provide a robust minimal genome reference for both ancestral genome reconstruction of the last common ancestor of extant sibling species^88^ and the bottom‐up design of a synthetic cell^87^.

Among 27,724 genes with COG annotations in five *Sinorhizobium* strains, enrichment analysis (Figure 5B and Data S1‐2; Fisher's exact test, *p* < 0.05) showed that these shared (quasi‐) essential genes were significantly enriched in the COG category J, M, H, U, F, and D. Shared 50 GD genes were enriched in K (transcription), P (inorganic ion transport and metabolism), and J (translation functions) (Supporting Information: Data S1‐3; Fisher's exact test, *p* < 0.05). Shared 1553 NE genes were enriched in S (unknown function), G (carbohydrate transport and metabolism), P, T (signal transduction mechanisms), and N (cell motility) (Supporting Information: Data S1‐4; Fisher's exact test, *p* < 0.05). There were 1599 genes shared by five strains, which exhibited a strain‐dependent requirement for growth (Supporting Information: Data S1‐5) and had an enrichment profile similar to that of shared (quasi‐)essential genes (Figure 5C; Fisher's exact test, *p* < 0.05).

In the pangenome context of 19 *Sinorhizobium* strains (Figure 1A,B), both subset I and II shared by five test strains showed strain‐dependent gene essentiality and intermingled ES‐GA categories (Figure S7A and Supporting Information: Data S1‐2, S1‐3, S1‐4). Gene function enrichment analysis showed that these shared (quasi‐)essential genes within subset I were significantly enriched in the COG category J, M, H, U, and D (Figure S7B and Supporting Information: Data S1‐2; Fisher's exact test, *p* < 0.05). These shared (quasi‐)essential genes within subset II were significantly enriched in the COG category J, M, and I (Figure S7B and Supporting Information: Data S1‐2; Fisher's exact test, *p* < 0.05). Furthermore, within the broader pangenome of 19 *Sinorhizobium* strains, the shared 50 GD genes were enriched in P, K, and J, which is identical with that of the GD genes within the pangenome background of the five test strains (Figure S8A and Supporting Information: Data S1‐3; Fisher's exact test, *p* < 0.05). And GD genes within subset I were enriched in P and J categories, while GD genes within subset II were enriched in K category (Figure S8B and Supporting Information: Data S1‐3; Fisher's exact test, *p* < 0.05). The fuzzy essential zone in *Sinorhizobium* pangenome highlighted differential roles of various COG categories in bacterial persistence (quasi‐essential, GD, and NE) under the nutrient‐rich condition (Figures 5B,C, S7, and S8).

**Figure 6:**
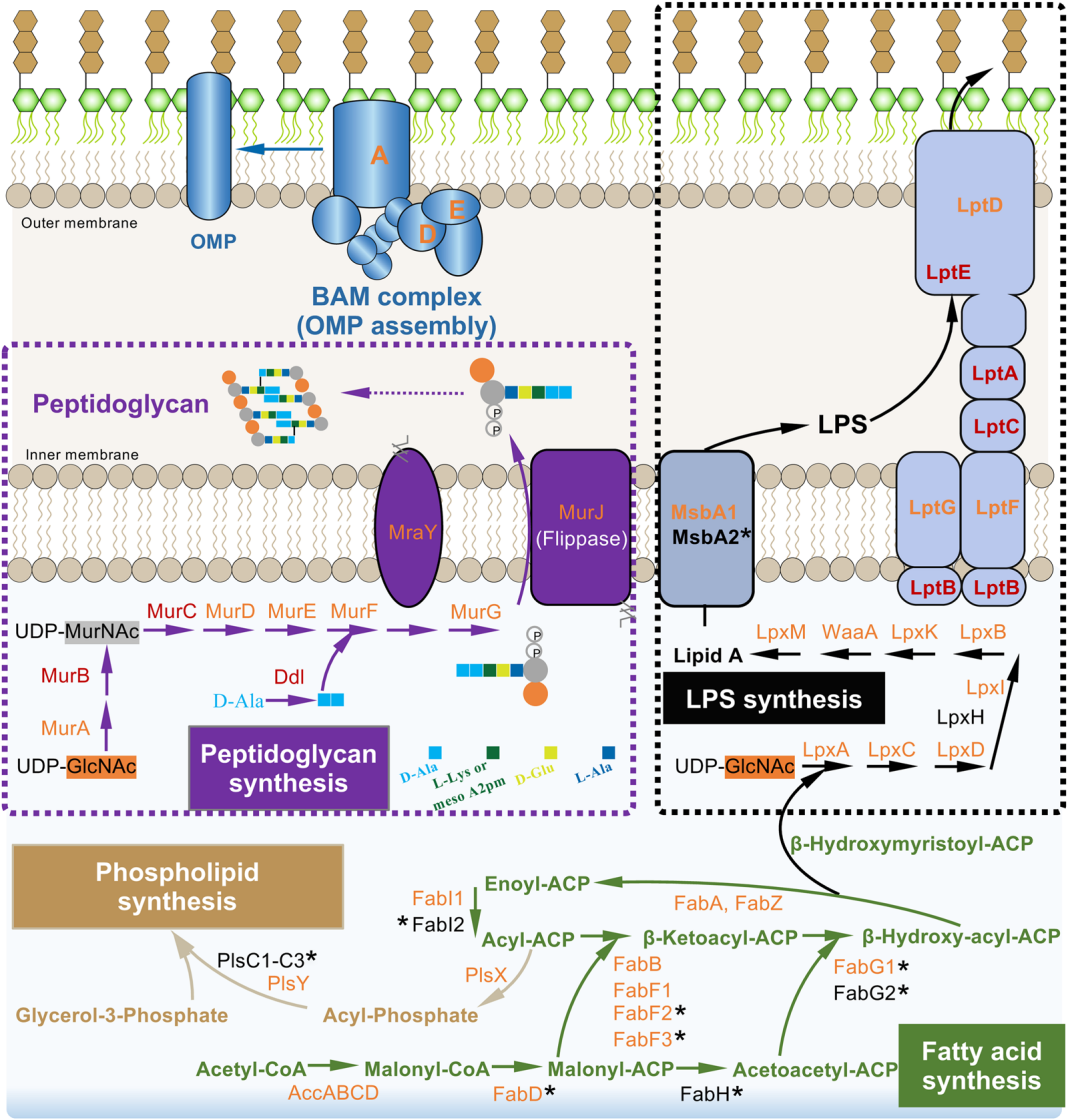
Cell envelope biogenesis is overrepresented in shared (quasi‐)essential genes and those showing strain‐dependent requirements for growth. Schematic view of cell envelope biogenesis machineries. Red, essential in all five strains; orange, either essential or growth‐advantage in all strains; orange proteins marked with *, either essential or growth‐advantage in all strains based on either HMM or Monte Carlo method; black proteins marked with *, required for strain‐dependent growth. ACC, acetyl‐CoA carboxylase; ACP, acyl carrier protein; BAM, complex involved in assembling various OMPs into the outer membrane; GlcNAc, N‐acetylglucosamine; LPS, lipopolysaccharide; MurNAc, N‐acetylmuramic acid; OMP, outer membrane protein.

Among the enriched COG categories in the cofitness network, no matter within the pangenome background of the five test strains or the broader pangenome of the 19 *Sinorhizobium* strains, it is noteworthy that J and M types of machinery were the top two functional categories over‐represented in shared (quasi‐)essential genes (Figures 5B and S7B), which is also the same for those showing strain‐dependent requirement for growth in the core genome of the five test strains (Figure 5C). Membrane proteins account for half of the cellular membrane mass and are hypothesized as a driver of the split between lipids of bacteria and archaea^89^, ^90^. Earlier in silico evidence also revealed that envelope proteins evolve faster than water‐soluble proteins^91^. In addition to envelope proteins, as shown in a schematic view of cell envelope biogenesis machineries in the cofitness network of *Sinorhizobium* (Figure 6), this portion of the fuzzy essential zone included genes involved in syntheses of fatty acids, phospholipids, lipopolysaccharides, and peptidoglycans, and assembly of outer membrane proteins. Phospholipids are essential components of cell membranes. Therefore, it is not unexpected that phospholipid synthesis genes, for example, *plsX* and *plsY*, were identified as (quasi‐)essential genes of *Sinorhizobium* (Figure 6). This is in line with the fact that *plsX* of *S. pneumoniae* has been proposed to be a new target for the development of antibacterial therapeutics^92^, ^93^. Lipopolysaccharides are important components of the outer membrane of Gram‐negative bacteria, and *lpxA*, *lpxD*, and *lptD* involved in lipopolysaccharide synthesis can be used as antibiotic targets^94^, ^95^, ^96^. These three and other genes involved in lipopolysaccharide synthesis were identified as (quasi‐)essential genes of *Sinorhizobium* (Figure 6). The fatty acid synthesis pathway provides precursors for lipopolysaccharide and phospholipid syntheses and was found to be (quasi‐)essential for *Sinorhizobium* (Figure 6). Similarly, fatty acid synthesis genes, for example, *accABCD*, *fabD*, *fabF*, and *fabG*, were also identified as ES core genes of human pathogen *Streptococcus pyogenes* ^97^. Peptidoglycan is a primary component of bacterial cell walls, and its synthesis pathway is the target of numerous antibiotics^98^. In this work, the peptidoglycan synthesis pathway was identified as (quasi‐)essential for *Sinorhizobium* (Figure 6). Similarly, peptidoglycan synthesis genes including *murB*, *murC*, *murD*, *murE*, *murF*, *murG*, *ddl*, and *mraY* belong to ES core genes of *S. pyogenes* in a Tn‐seq study^97^. Among these peptidoglycan synthesis genes, *murG* required for synthesizing the peptidoglycan precursor Lipid II^99^ is also essential for *Staphylococcus aureus* ^29^, and *murJ* encoding peptidoglycan lipid II flippase is indispensable for the viability of *Burkholderia cenocepacia* ^100^, ^101^. Collectively, these findings underscore the indispensability of bacterial cell envelope. All of these envelope‐related functions can be found in the predicted last bacterial common ancestor^86^. Membranes are the boundary between a cell and its biotic/abiotic surroundings, directly involved in adaptations to new niches and genetic material exchange^9^, ^102^, ^103^. Therefore, network analysis of the pangenomic Tn‐seq can also provide functional insights into bacterial evolutionary mechanisms.

In summary, based on a robust Tn‐seq analysis of independent *mariner* transposon insertion libraries of *Sinorhizobium* strains (Figures 1 and 2), pangenomic and network‐based analyses (Figures 1B, 2, 3, 4) allowed identification of a strain‐dependent variation in the fitness network (harboring ES, GA, GD, and NE genes) of *Sinorhizobium* pangenome under a nutrient‐rich condition. This fitness network is characterized by a highly connected ES subnetwork and beneficial (GA) and deleterious (GD) subnetworks of lower connectivity (Figure 4). Genus core genes belonging to both the shared and strain‐dependent essential zones of this fitness network exhibited a similar profile of functional categories, for example, cell envelop biogenesis (Figures 5 and 6). The network‐based analyses of the fuzzy essential zone of *Sinorhizobium* pangenome developed in this work can be used for any prokaryotes for which a robust Tn‐seq procedure can be established. These efforts are significant for fully understanding the evolution of prokaryote pangenome, the in silico bipartition of which into ES core and NE accessory subsets is oversimplified.

## MATERIALS AND METHODS

### Bacterial strains and growth conditions

The bacterial strains and plasmids used in this study are summarized in Data S2. *Sinorhizobium* strains were cultured in TY medium^63^ (5 g l^−1^ tryptone, 3 g l^−1^ yeast extract, and 0.6 g l^−1^ CaCl_2_) supplemented with 30 µg ml^−1^ nalidixic acid (NA) and 10 µg ml^−1^ trimethoprim (Tmp) at 28°C. *Escherichia coli* strains harboring vector pSAM_Sf were grown in Luria‐Bertani (LB) medium^104^ at 37°C supplemented with 50 µg ml^−1^ carbenicillin (Cb) and 50 µg ml^−1^ kanamycin (Km).

### Construction of pSAM_Sf and transposon insertion library preparation

The mariner‐based transposon suicide delivery vector pSAM_Sf was retrofitted from a previously described *Mme*I‐adapted mariner delivery vector pSAM_Bt^61^. Briefly, the original *Bacteroides thetaiotaomicron rpoD* promoter region was replaced with the *rpoD* promoter fragment amplification using primers PropD‐F/PropD‐R (Supporting Information: Data S2) from *S. fredii* CCBAU45436, and the original erythromycin resistance gene *ermG* was replaced with the Km resistance gene by PCR amplification using primers kan‐F/kan‐R (Supporting Information: Data S2) from pRL1063a^62^. The resulting transposon mutagenesis vector pSAM_Sf was then transferred into *E. coli* S17‐1 λpir to obtain a donor strain *E. coli* strain S17‐1 λpir/pSAM_Sf. The resulting transposon mutagenesis vector pSAM_Sf was then transferred into each *Sinorhizobium* strain for creating a mutant library using bi‐parental mating. Three independent mutant libraries for each strain were constructed and collected. Specifically, each of the five wild‐type *Sinorhizobium* strains (recipient strain) and the donor strain were individually grown to late exponential phase (OD_600_ = 1.2–1.4), and then each recipient strain and donor strain were transferred and diluted to 1:200 in fresh TY and LB medium for further culture to mid‐log phase (OD_600_ = 0.6–0.7), respectively. Each bacterial culture with the defined optical density was firstly pelleted at 8000 rpm for 3 min in 50‐ml centrifuge tubes, washed once with 50 ml NaCl solution (0.85%, wt/vol), and then mixed in a 2:1 ratio of each recipient strain and donor strain, and each of the five rhizobia‐*E. coli* mixtures was centrifuged and spotted dropwise (50 μl) onto a TY agar plate without any antibiotics for plasmid transfer. The mating plates were incubated at 28°C for 36 h. Each resulting transconjugant mixture was then resuspended in 1 ml NaCl solution (0.85%, wt/vol) and spread equally (100 μl mixture) onto each TY agar plate supplemented with NA, Tmp and Km antibiotics (*S. meliloti* 2011 rather than the remaining *Sinorhizobium* strains needed fivefold concentration of Km for mutant screen since the wild‐type strain could tolerate 50 µg ml^−1^ of Km antibiotics) and incubated at 28°C to obtain mutants represented by single colonies. Mutant libraries for each strain were individually scraped and collected to do subsequent genomic DNA extraction.

### Tn‐Seq sample preparation for sequencing

Genomic DNA from individual mutant libraries was extracted using a TIANamp bacteria DNA kit (TIANGEN). Then 3.5 µg of gDNA was digested with 3 µl of *Mme*I (New England Biolabs) for 2.5 h at 37°C and further treated for 1 h with 2 µl of calf intestinal alkaline phosphatase (CIP) (New England Biolabs). Double‐stranded adapter DNA with distinct 12‐bp barcode (Supporting Information: Data S2) was prepared by mixing single‐stranded adapter pair (a final concentration of 0.2 mM for each adapter) in 1 mM Tris·Cl (pH 8.3). This reaction mixture was incubated at 95°C for 5 min and then allowed to slowly cool down (0.1°C/s). Double‐stranded adapter molecules were ligated to *Mme*I‐digested gDNA in a T4 DNA ligation reaction mixture (New England Biolabs) harboring 25 µl of gDNA, 3 µl of T4 DNA ligation buffer, 1 µl (400 U/μl) of T4 DNA ligase, and 1 µl of 0.1 mM double‐stranded adapter. The resulting *Mme*I‐digested gDNA with ligated adapter (2 µl) as DNA template was then PCR amplified with 22 cycles using Q5 High‐Fidelity DNA polymerase (New England Biolabs) according to the manufacturer's instructions. All PCR products were subject to electrophoresis on a 1.8% (wt/vol) agarose gel, and the 142‐bp DNA bands were purified from the excised gel slices using the QIAquick Gel Extraction Kit (Qiagen). The universal transposon primer and adapter primer were used for each PCR reaction (Supporting Information: Data S2). These primers contained necessary anchor sequences for annealing to oligos present in the flow cell. Tn‐seq was performed on three independently generated libraries for each strain. Single‐end sequencing was performed on the NextSeq 550AR platform (Annoroad Gene Technology Co., Ltd.) using the sequencing primer as shown in Supporting Information: Data S2.

### Gene essentiality analysis of Tn‐seq data

HMM^55^, Bayesian^56^, and Monte Carlo^57^ methods were used to define ES genes. Briefly, the raw reads were first filtered by using fqgrep (https://github.com/indraniel/fqgrep) to identify the adapter or transposon sequence, and then the genomic DNA (gDNA) (16–17 bp) adjacent to each transposon was extracted. The resulting sequences after extraction were aligned to the reference genomes of individual strains using bowtie 2^105^, allowing for a 1‐bp mismatch in the alignment, resulting in a*.sam* output file. The extracted reads mapped to the extreme 5′ and 3′ ends of genes (5% of each end) were excluded from further analysis to minimize the potential effect of nondisruptive insertions^31^. The number of reads with a leading “TA” motif mapped to the genome of each strain was counted, and the number of transposon insertion sequences inserted into the TA site was subsequently calculated. Bayesian‐^56^ and HMM‐^55^ based methods have been integrated in the TRANSIT software^66^. A *.wig* format file of the aligned gDNA that can be recognized by the TRANSIT software^66^ was generated from the *.sam* format file using a Python script (summarize_mappings.py, https://github.com/elijweiss/Tn-seq). The data were then smoothed by using locally weighted LOESS regression and normalized by using the TTR (trimmed total reads) method with default parameters in TRANSIT software^66^. ES genes were identified by Bayesian‐ and HMM‐based methods with default parameters. The HMM‐based method also assigns GD, GA, and NE states to genes^66^. For the Monte Carlo method^57^, 2000 “Expected” pseudodatasets were generated by randomly assigning the read counts from all insertion events to all available TA sites in the genome. Then differential mutant abundance between the “Observed” data set and the “Expected” pseudodatasets (fitness value) was calculated as log_2_(Fold change) using DESeq2 package^106^. ES genes were identified for those with log_2_(Fold change) < −1 (adjusted. *p* < 0.05).

### Bioinformatic procedures in *Sinorhizobium* pangenome analysis

Homologous genes among *Sinorhizobium* strains were identified by OrthoFinder with default parameters (‐M msa ‐a 40)^107^. Using the 1667 core genes shared by 19 *Sinorhizobium* strains, and *Ensifer adhaerens* Casida A, a species phylogenetic tree (maximum likelihood) was constructed using RaxML^108^ with the PROTGAMMAAUTO setting using 250 bootstrap replicates. Pangenome subsets of *Sinorhizobium* strains were defined as follows: Subset I, genes shared by 19 strains; Subset II, genes shared by 2 to 18 strains; Subset III, strain‐specific genes. COG, GO, and KEGG annotation information for all genes of five *Sinorhizobium* strains was determined by the eggNOG 4.5 database^109^. Based on the fitness values generated by the Monte Carlo method, Pearson's correlation coefficient between shared genes of five strains was calculated, resulting in a 3284 × 3284 gene versus gene matrices. The network correlation threshold (Pearson's *r* > 0.91) was detected by the RMT‐based approach^75^, ^76^, ^77^, ^78^. The retained gene pairs were used as edges to construct the cofitness network consisting of 3284 genes and 228,134 edges. The cofitness network analyses were carried out using an igraph R package^110^ and Cytoscape 3.7.0^111^, including network metrics such as connectivity degree (which reflects the quantity of links a node has to others), average shortest path length (denoting the mean steps required to traverse the shortest paths between all node pairs), closeness centrality (derived from the inverse of the aggregate shortest path lengths from a specific node to every other node within the graph), and the topological coefficient (evaluating the extent to which a node shares its neighbors with other nodes). Tukey HSD test for fitness comparison and the cofitness network metrics analysis were carried out using the agricolae R package^112^. Gene function enrichment analysis was carried out using Fisher's exact test, employing the standard R function “fisher.test”. Additionally, enrichment analysis was performed for the proportion of subset I‐III genes belonging to ES, GA, GD and NE categories by Fisher's exact test and *Z* test. The *Z* test calculates the *z*‐score value through 5000 random simulations, thereby determining the *p* value (two‐tailed) modeled on a Gaussian distribution^113^.

## AUTHOR CONTRIBUTIONS

**Pan Zhang:** Data curation (equal); formal analysis (equal); investigation (equal); validation (equal); writing—original draft (equal); writing—review and editing (equal). **Biliang Zhang:** Data curation (equal); methodology (equal); software (equal); writing—original draft (equal); writing—review and editing (equal). **Yuanyuan Ji:** Investigation (supporting). **Jian Jiao**: Investigation (supporting). **Ziding Zhang:** Methodology (equal); software (equal); supervision (equal). **Chang‐Fu Tian:** Conceptualization (lead); funding acquisition (lead); methodology (equal); resources (lead); supervision (lead); writing—review and editing (equal).

## ETHICS STATEMENT

No animals or humans were involved in this study.

## CONFLICT OF INTERESTS

The authors declare no conflict of interests.

## Supporting information


**Data S1.** Summary of Tn‐seq analysis.

**Data S2.** Strains, plasmids, and primers used in this study.

Supporting information.

Supporting information.

Supporting information.

Supporting information.

Supporting information.

Supporting information.

Supporting information.

Supporting information.

## Data Availability

The Tn‐seq data underlying this article are available in GenBank Database at https://www.ncbi.nlm.nih.gov/bioproject, and can be accessed with BioProject ID PRJNA699738. Genome annotation information used in this work is shown in Data S1.
