# Supplementary material for: Cofitness network connectivity determines a fuzzy essential zone in open bacterial pangenome
Source: mLife. 2024 Jun 28;3(2):277–90. doi: 10.1002/mlf2.12132 (PMC11211677; doi:10.1002/mlf2.12132)
Supplement: Supplementary file 3 — Supporting information. [file MLF2-3-277-s002.pdf]

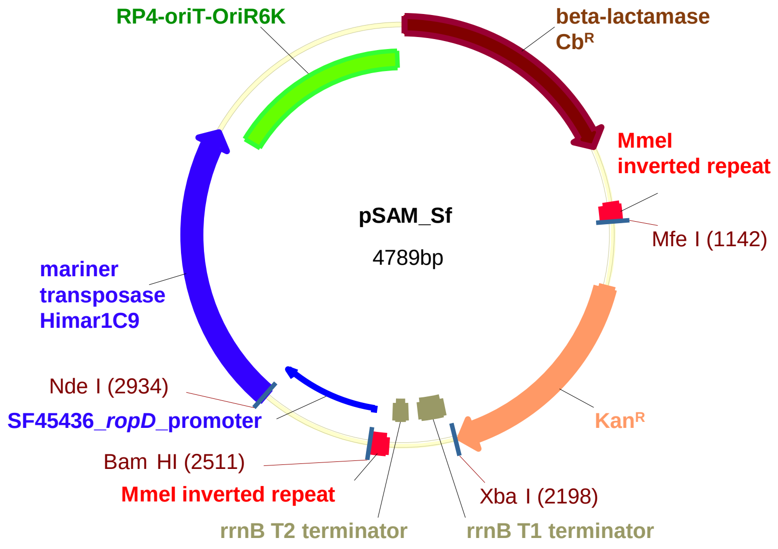


**Figure S1. Construction of the *mariner* transposon carrying pSAM_Sf for *Sinorhizobium* species**. pSAM_Sf derived from pSAM Bt carrying the *mariner* transposase Himar1C9. The *ermG* gene was replaced by kanamycin resistance gene cloned from the plasmid pRL1063a and a *rpoD* promoter of *S. fredii* CCBAU45436 was inserted to drive expression of the Himar1C9 transposase within *Sinorhizobium* strains. Plasmid map was drawn by Vector NTI software.
