# Supplementary material for: Cofitness network connectivity determines a fuzzy essential zone in open bacterial pangenome
Source: mLife. 2024 Jun 28;3(2):277–90. doi: 10.1002/mlf2.12132 (PMC11211677; doi:10.1002/mlf2.12132)
Supplement: Supplementary file 4 — Supporting information. [file MLF2-3-277-s009.pdf]

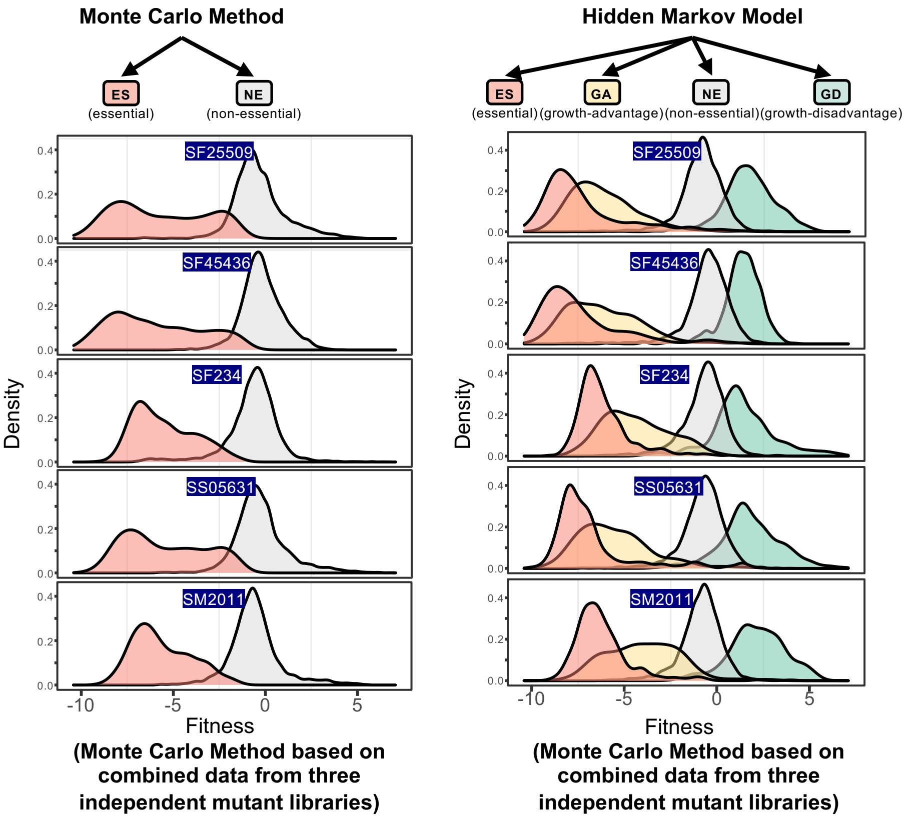

Figure S2. Density of fitness values calculated by Monte Carlo method for ES and NE defined by Monte Carlo method (left), and for ES, GA, NE, and GD genes defined by HMM method (right). The vertical axis showing distribution of fitness values for genes assigned as ES (essential), GA (growth-advantage), NE (non-essential) and GD (growth-disadvantage) in five strains. Combined data from three independent mutant libraries for individual strains were analyzed by using both Monte Carlo and HMM methods.
