# Supplementary material for: Cofitness network connectivity determines a fuzzy essential zone in open bacterial pangenome
Source: mLife. 2024 Jun 28;3(2):277–90. doi: 10.1002/mlf2.12132 (PMC11211677; doi:10.1002/mlf2.12132)
Supplement: Supplementary file 5 — Supporting information. [file MLF2-3-277-s003.pdf]

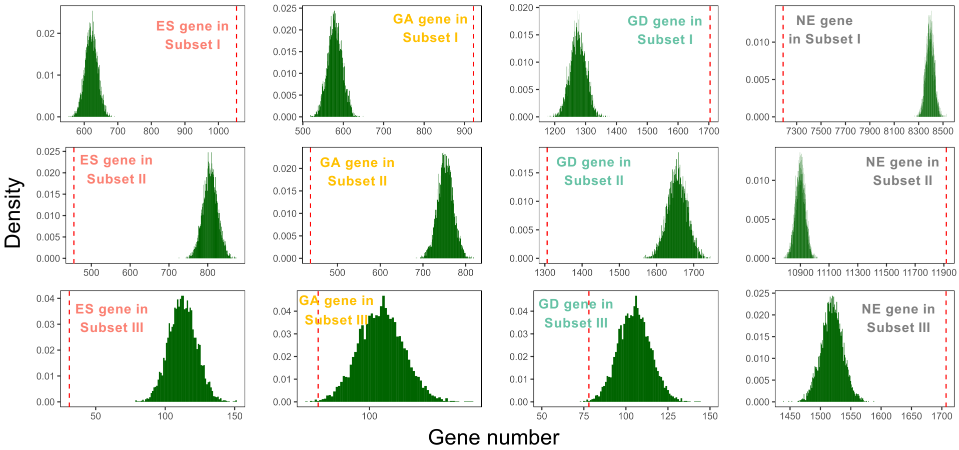

Figure S3. Enrichment analysis of ES/GA/GD/NE genes in subset I-III. The same number of genes as subset I-III were randomly selected, and the y-axis represents the density distribution of the number of genes belonging to ES/GD/GA from 5000 random extractions, and the red dotted line represents the true gene number. Based on the Z test, it was found that all the true gene numbers here were significantly different compared to the random results (*P* value < 0.01).
