# Supplementary material for: Cofitness network connectivity determines a fuzzy essential zone in open bacterial pangenome
Source: mLife. 2024 Jun 28;3(2):277–90. doi: 10.1002/mlf2.12132 (PMC11211677; doi:10.1002/mlf2.12132)
Supplement: Supplementary file 6 — Supporting information. [file MLF2-3-277-s008.pdf]

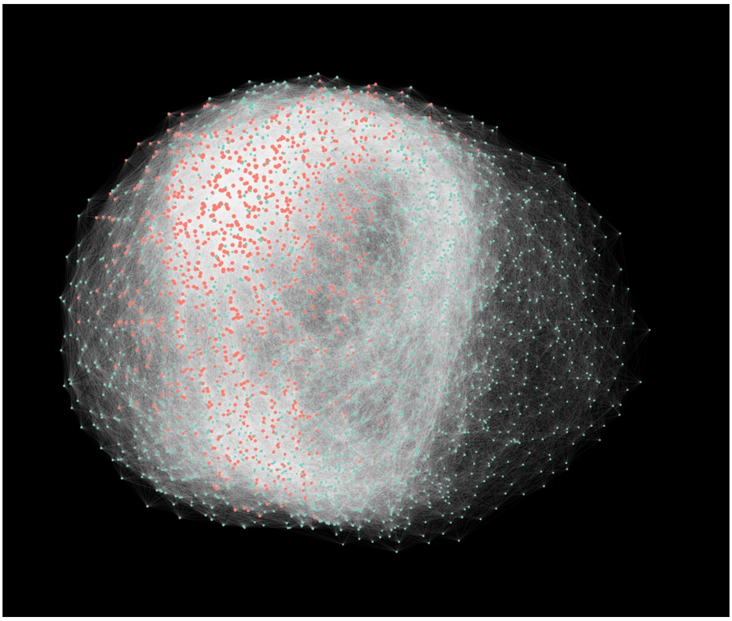

Figure S4. The co-fitness network analysis of Monte Carlo method-based fitness values. Fitness value of 3,284 core genes among the five test strains were used to create the co-fitness network, red dots indicate genes in Module_1, which is located in the center of the network.
