# Supplementary material for: Cofitness network connectivity determines a fuzzy essential zone in open bacterial pangenome
Source: mLife. 2024 Jun 28;3(2):277–90. doi: 10.1002/mlf2.12132 (PMC11211677; doi:10.1002/mlf2.12132)
Supplement: Supplementary file 7 — Supporting information. [file MLF2-3-277-s004.pdf]

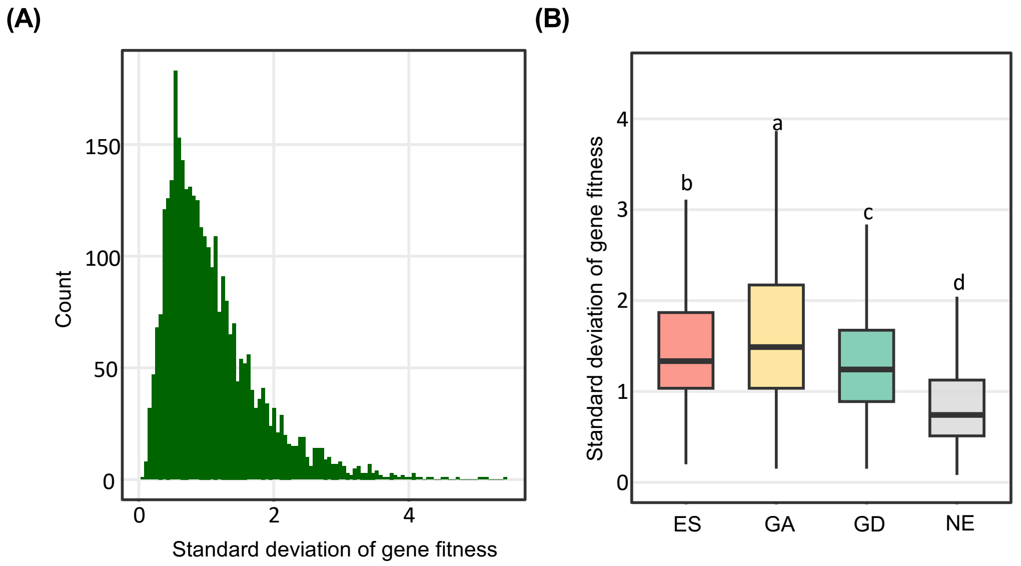

Figure S5. Standard deviation of Monte Carlo method-based fitness values among five strains. (**A**) Distribution of standard deviation (SD) for fitness values of 3,284 genes, which conforms to normal distribution. (**B**) The SD values for gene fitness values vary among genes of different categories. Different letters indicate significant differences between means (Tukey HSD test, adj.*P* value < 0.05).
