# Supplementary material for: Cofitness network connectivity determines a fuzzy essential zone in open bacterial pangenome
Source: mLife. 2024 Jun 28;3(2):277–90. doi: 10.1002/mlf2.12132 (PMC11211677; doi:10.1002/mlf2.12132)
Supplement: Supplementary file 8 — Supporting information. [file MLF2-3-277-s001.pdf]

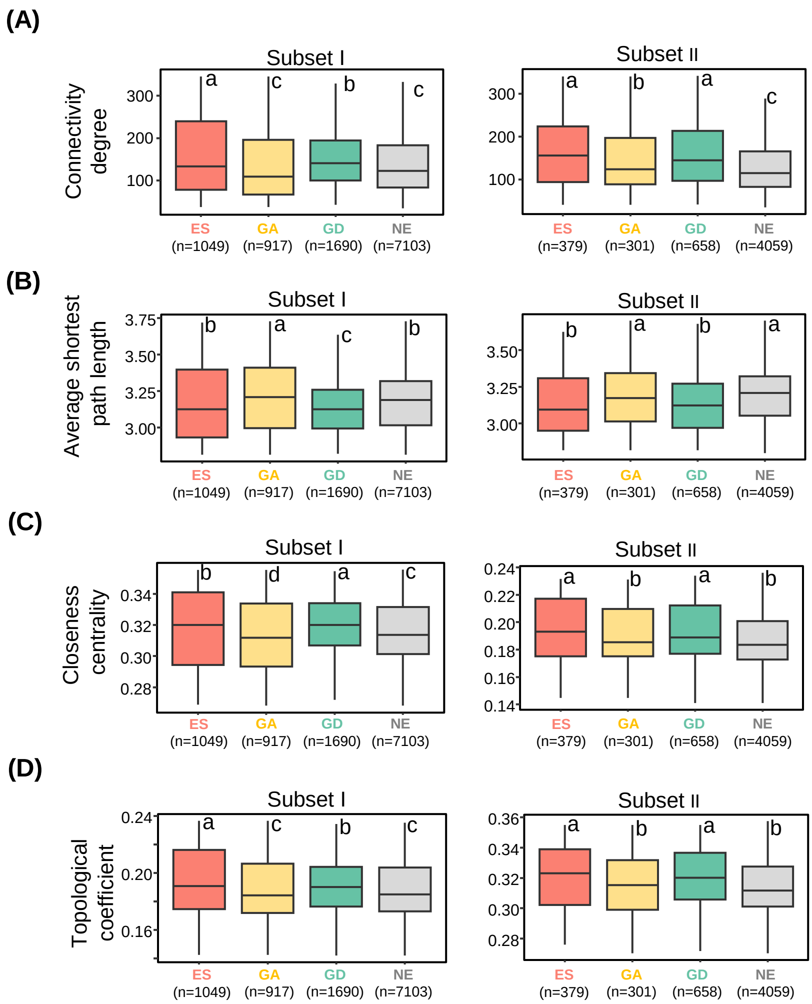

Figure S6. The co-fitness network features were evaluated for subset I and II. Network analysis of genes with different conservative levels, including connectivity degree (**A**), average shortest path length (**B**), closeness centrality (**C**) and topological coefficient (**D**). Different letters indicate significant differences between means (Tukey HSD test, adj.*P* value < 0.05).
