# Supplementary material for: Cofitness network connectivity determines a fuzzy essential zone in open bacterial pangenome
Source: mLife. 2024 Jun 28;3(2):277–90. doi: 10.1002/mlf2.12132 (PMC11211677; doi:10.1002/mlf2.12132)
Supplement: Supplementary file 9 — Supporting information. [file MLF2-3-277-s007.pdf]

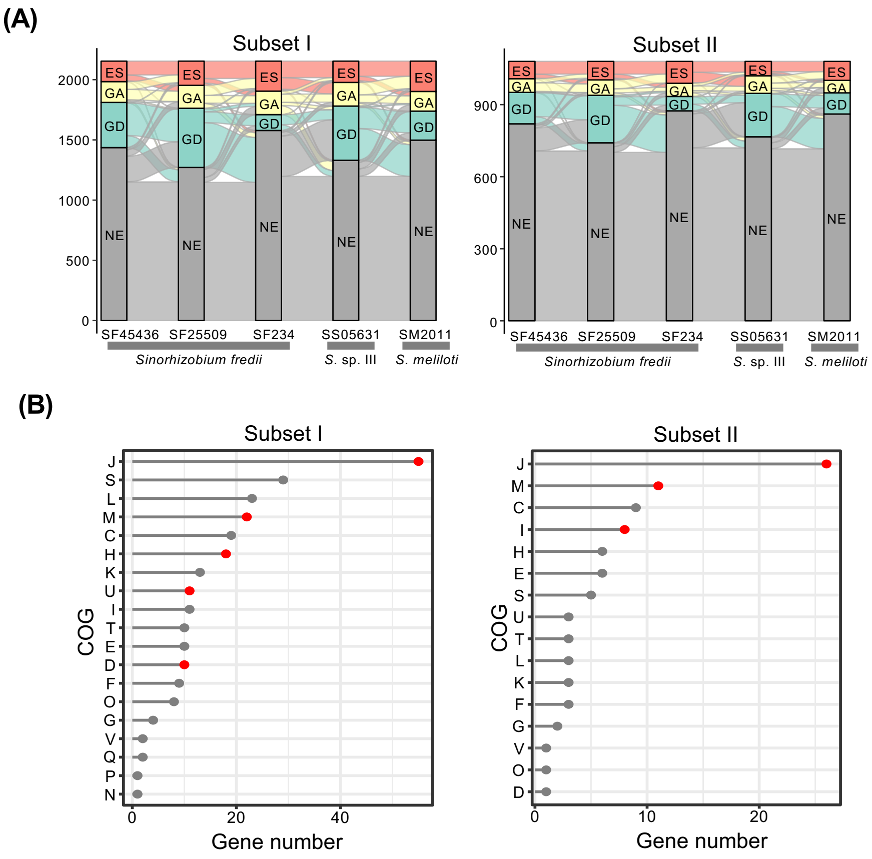

Figure S7. COG enrichment analysis of shared (quasi-)essential genes in different conservation levels. (**A**) Among the genes shared by the five test strains, 2154 genes belong to subset I, 1081 genes belong to subset II. (**B**) COG enrichment analysis of shared (quasi-)essential genes in subset I and subset II. Red dots in (**B**), $P < 0.05$ in Fisher's exact test.
