# Supplementary material for: Cofitness network connectivity determines a fuzzy essential zone in open bacterial pangenome
Source: mLife. 2024 Jun 28;3(2):277–90. doi: 10.1002/mlf2.12132 (PMC11211677; doi:10.1002/mlf2.12132)
Supplement: Supplementary file 10 — Supporting information. [file MLF2-3-277-s010.pdf]

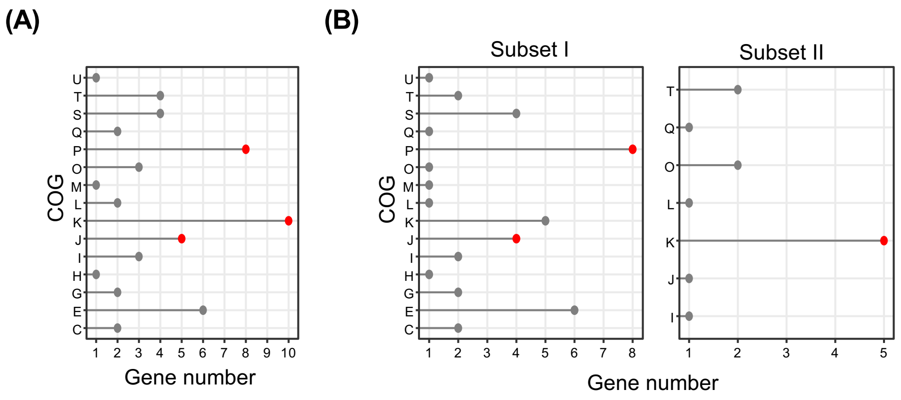

Figure S8. COG enrichment analysis of shared GD genes in different conservation levels. (**A**) COG enrichment analysis of all shared GD genes (50 genes). (**B**) COG enrichment analysis of shared GD genes in subset I and subset II. Red dots indicate $P < 0.05$ in Fisher's exact test.
